# The spatial frequency domain designated watermarking framework uses linear blind source separation for intelligent visual signal processing

**DOI:** 10.3389/fnbot.2022.1054481

**Published:** 2022-11-11

**Authors:** Rani Kumari, Abhijit Mustafi

**Affiliations:** Department of Computer Science, Birla Institute of Technology, Ranchi, India

**Keywords:** digital watermarking, fractional Fourier transform, blind source separation, genetic algorithm, robustness

## Abstract

This paper develops a digital watermarking algorithm using an informed watermark retrieval architecture. The developed method uses the fractional Fourier transform to embed the watermark in the space-frequency domain and extracts the watermark using blind source separation techniques. The watermark embedding is further enhanced using a heuristic algorithm to increase the strength of the watermarking system. We use genetic algorithm to find the optimal fractional domain by minimizing the coefficient of RMSE between the input image and the watermarked image. The algorithm's performance against various common attacks, e.g., JPEG compression and Gaussian noise, is presented to estimate the algorithm's robustness.

## Introduction

With the proliferation of computers and other digital devices in society, the rapid growth of the internet, the demand for free availability of copyrighted digital entities, and weak cyber laws existing in most nations, digital piracy has grown to massive proportions in the past few years. Authorities worldwide are struggling to contain this phenomenon, costing many industries a vast amount of money in terms of revenue. Even though many systems have been developed to fight this menace, the effectiveness of these systems remains in question for large-scale public use. Many of these systems utilize the tenets of cryptography to encrypt digital entities before distributing them across networks (public key and private key infrastructures). However, such systems are helpless if rightful owners of copyrighted digital entities make illegal copies of the material and proceed to distribute it illegally. Cryptographic systems are quite capable of preventing abuse of digital entities during the distribution phase but are not very effective once the decryption process has been performed.

Watermarking and steganographic systems attempt to fill this void and complement the functioning of cryptographic algorithms. These algorithms control the authenticity of digital entities even after decryption and allow the ownership of digital entities to be verified. This can be of extreme importance not only for public use but also in sensitive application domains like defense and intelligence. Many of these domains need to maintain stringent control on distributed digital entities, and verifying ownership is crucial. Establishing a source-verified or informed watermarking system is considered an extremely efficient solution in such cases, and developing such systems has become an area of active research.

Various methods have been cited in the literature to embed watermarks in signals. These techniques can be broadly categorized as time (space) domain techniques and frequency domain techniques. For digital images, spatial domain watermarks are embedded by directly manipulating the intensity values of the individual pixels to cause little change in the visual perceptibility of the image. Methods in this category include LSB (least significant bit) based techniques, XOR-based techniques, color manipulation, etc. In the frequency domain, the image is first transformed to the frequency domain using the Fourier transform before the transform coefficients are manipulated to embed the watermark. Watermarking techniques have recently been developed in the time-frequency domain and using the fractional Fourier transform. Wavelet-based techniques have also been proven to be highly effective in digital watermarking. The efficiency of spread spectrums in channel communication has also drawn the attention of researchers working in watermarking and steganography. Spread spectrums provide an excellent diffusion mechanism for spreading watermark signatures across a random set of frequencies and be extremely robust.

In this paper, we present the design of a watermarking system for digital images using the fractional Fourier transform (FrFT) and blind source separation (BSS). The developed system embeds a watermark in the space-frequency domain, and the retrieval of the watermark is accomplished using a BSS technique. The embedded watermark is inserted using a mixing matrix whose coefficients are optimized using genetic algorithms to ensure that the watermark is not visually perceivable and the integrity of the pixels in the watermarked image is minimally compromised in comparison to the original image.

## Related work

In recent decades a digital revolution has occurred that no one could have imagined a few years ago. Massive digitalization has revolutionized the way we work and our social interactions (Woods and Gonzalez, 2002). While digitalization has made data easier to store and preserve, it has also posed cognitive issues of global significance, requiring massive computational infrastructure and efficient algorithms. The storing of textual documents in digital archives is a case in point. The prevailing trend was to scan text documents as images and submit them to storage repositories.

Now, we have tools and techniques that allow us to create digital texts that can be encoded using ASCII and UTF-8/16 (Djurovic et al., 2001). Scanned text documents are convenient to store but processing them is difficult. Text extraction from scanned images is not harrowing, but it is far from perfect. The massive production and storage of digital documents is an issue for organizations worldwide. The internet's advent has created new pathways for generating virtual text, with the web offering several alternatives (Cox et al., 2008). Web pages, social media sites, encyclopedias, and other internet sources are widespread. If new digital techniques are not investigated, the speed at which documents are produced could surpass our computational capabilities. In this paper, we investigate another aspect of the digital revolution which has to do with security in the form of watermarking.

A watermarking technique for identifying copyright infringement was developed by Komatsu and Tominaga (1989), specifically for digital entities. The idea of storing a watermark generated with spread spectrum methods and matrix transformations that was imperceptible in grayscale images and resistant to tempering was given by Boland et al. (1995). It has suggested a technique for embedding robust watermarks in images. Recent years have seen the development of watermarking methods in both frequency and spatial domains. Several methods have been used in spatial domain algorithms, including paired pixel manipulation, LSB substitution in the host images, and textured block coding. LSB substitution and several variations are the most effective and computationally efficient methods for embedding watermarks (Lu et al., 2003). However, LSB replacement is still not considered a robust algorithm, notwithstanding these attempts to improve it. A secure spatial domain technique was devised by Lin (2000) to survive challenging attacks, including JPEG compression.

The frequency-domain digital watermarking algorithms offer significantly more security than their spatial counterparts. The algorithms transform images from the time domain to the transform domain and then embed the watermark into the frequency domain (Abraham and Paul, 2017). Many of these methods involve the discrete Fourier transform (Candan et al., 2000), discrete cosine transform (Hernandez et al., 2000), or discrete wavelet transform (Xia et al., 1998). Zhang et al. (2020) effectively encrypted color images using a 2D discrete Fourier transform and a blind watermarking method. Fares et al. (2020) proposed a method for color images in the Fourier transform domain where embedding was carried out independently in each image plane. The discrete wavelet transform was effectively employed by Xia et al. (1997) to embed watermarks in multiresolution images. In this method, the significant coefficients in the high and medium frequency bands of the DWT image were assigned pseudo-random codes.

The fractional Fourier transform, and its applications have been explored extensively by researchers. One of the most well-known early introductions to the transformation is given by Namias (1980). He also suggested embedding watermarks in images using the technique of phase shift keying to make the watermarking more imperceptible. Kumar et al. (2013) created a blind digital image watermarking system based on the FrFT. The study proved that the FrFT could be used to provide good imperceptibility and resilience to complex JPEG compression attacks. Based on the FrFT, Mustafi and Ghorai (2013) proposed a new method for denoising medical images. The presented method uses blind source separation and fractional Fourier transform algorithm to eliminate noise from medical images, resulting in enhanced and robust denoising. Lang and Zhan (2014) proposed a new blind image watermarking method based on the FrFT for embedding a visually unidentifiable watermark into an image. The original cover image is divided into non-overlapping blocks for watermarking, each modified using a 2D fractional Fourier transform of two fractional orders. Kumari and Mustafi (2020) presented a straightforward digital watermarking method based on the fractional Fourier transform. This presented work provides a more secure information hiding technique that is robust, undetectable and has a more extensive data concealing capacity to meet the needs of each recipient across a broad spectrum of frequencies and spatial domains. Kumari and Mustafi (2021) developed a powerful image watermarking method based on the 2D discrete fractional Fourier transform. The method includes a twofold transform technique to improve its robustness to attacks. PSO was used to determine the best fractional ordering for embedding watermarks in the cover image. Kumari and Mustafi (2022) described an effective solution for image denoising using the FrFT. Images are denoised using a parallel set of filters and FrFT to extract the watermark. Simulations show that their approach is as accurate as current denoising techniques.

Recent watermarking techniques have also used nature-based algorithms (Naheed et al., 2014). Most watermarking techniques require lengthy searches to determine the ideal location for the watermark (in space or frequency domain). Due to local optima and high dimensionality, standard search strategies are often inadequate. Shieh et al. (2004) proposed a genetic algorithm-based watermarking approach in the transform domain. GA optimizes conflicting requirements. Watermarking using GA seems to be simple. They also evaluate their approach using GA's fitness function, which considers robustness and invisibility. Simulations illustrate GA's robustness under attacks and improvement in watermarked image quality. Wang et al. (2011) provided an optimal image watermarking methodology employing a multiobjective genetic algorithm in accordance with the multiobjective nature of image watermarking. A multiobjective genetic algorithm was employed to autonomously determine the optimal watermarking parameters, and a variable-length mechanism was used to seek the best watermark embedding locations. The optimization results indicate that multiobjective watermarking can increase the performance of watermarking algorithms without the problem of determining optimal parameters. Naheed et al. (2014) devised reversible watermarking to enhance embedding strength and invisibility. GA and PSO-based reverse interpolation watermarking provide for medical and standard images. Experimental data show that the suggested technique improves perceptual quality and the size of the watermark payload. An effective blind digital watermarking system based on a genetic algorithm is given by Alvarez et al. (2018). The experimental results suggest that, compared to previous approaches in the literature, the scheme maintains invisibility, security, and robustness more frequently. Calculations showed that the proposed watermarking method is robust to several attacks caused by salt and pepper, Gaussian noise, and jpeg compression.

Blind source separation (BSS) methodologies are used to separate audio, image, or any other source signal from a group of observation or mixed signals without identifying the mixing procedure and source signal characteristics (Sanchez, 2002). Separation is performed using several algorithms. However, BSS employing Non-Negative Matrix Factorization is frequently used. Non-Negative Matrix Factorization algorithms (Silva et al., 2020) still have difficulties with solution space convergence and separation quality. BSS was described by Belouchrani et al. (1997) as the recovery of a set of sources from a mixture without knowledge of the original signals or mixing technique. When a single source is recovered from several mixtures, the problem is called blind source separation (Choi et al., 2002). Recent studies by Silva et al. (2020) developed an output-only operational modal analysis method based on blind source separation. The method uses each pixel as a measurement point. This increases sensor density by orders of magnitude. Using extracted modal data, a simple method is provided to magnify and visualize independent vibration modes. The results show that the proposed technique can decompose, visualize, and rebuild weakly stimulated vibration modes.

## Overview of fractional Fourier transform and blind source separation

The fractional Fourier transform coupled with BSS can provide an extremely efficient framework for developing watermarking and steganographic systems. The FrFT, with its ability to provide a singular domain space-frequency representation of a signal (Ozaktas et al., 2001), can quickly disperse an embedded watermark in the host entity to prevent localization attacks in the spatial domain. Such watermarks are usually challenging but simple to retrieve using blind source separation. The algorithm's simplicity is enhanced because BSS does not require any apriori knowledge at the retrieval end to extract the watermark. Consequently, the watermarking system is not only extremely robust but also simple to operate for the authorized user.

### Fractional Fourier transform

The fractional Fourier transform (FrFT) is an extension of the Fourier transform (FT) with an additional degree of freedom α(0 ≤ α ≤ 1), known as the order of the transform (Namias, 1980). This extra degree of freedom allows the FrFT to generate a powerful time (spatial) frequency signal representation. The FrFT has been compared to the short-term Fourier transform, Wigner Distribution, and wavelets in literature. As FrFT is a specialization of the Fourier transforms, it is also reversible and follows Parseval's theorem (Mustafi and Ghorai, 2013). Though not as closed and mathematically consistent as its analog counterpart, the discrete and two-dimensional version of the transform also exists under moderate limitations that are almost always satisfied.

The formal definition of the FrFT employs a forward transformation kernel *K*_α_, which is defined in Eq. (1) (Bultheel and Sulbaran, 2004).


(1) $$K_{\alpha }\left(t,u\right)=\{\begin{array}{c} \begin{array}{c} \delta \left(t-u\right)\;\alpha \;is\;a\;multiple\;of\;2\pi \\ \;\\ \delta \left(t+u\right)\;\left(\alpha +\pi \right)is\;a\;multiple\;of\;2\pi \\ \sqrt{\frac{1-jcot\left(\alpha \right)}{2\pi }}e^{j\left(\frac{u^{2}+t^{2}}{2}\right)\cot\left(\alpha \right)-j\;\text{ut}\;\text{cosec }\left(\alpha \right)\;}\;else \end{array}\; \end{array}$$

Using this forward transformation kernel, the FrFT of order α is defined in the regular form as


(2) $$F_{\alpha }\left(u\right)=\int_{-\infty }^{\infty }f\left(t\right)K_{\alpha }\left(t,u\right)dt$$

The Euler representation, which is used to further simplify the expression in Eq. (1)


(3) $$\sqrt{\frac{1-jcot(\alpha )}{2\pi }=}\sqrt{\frac{-je^{j\alpha }}{2\pi \;sin(\alpha )}}$$

**Figure 1:**
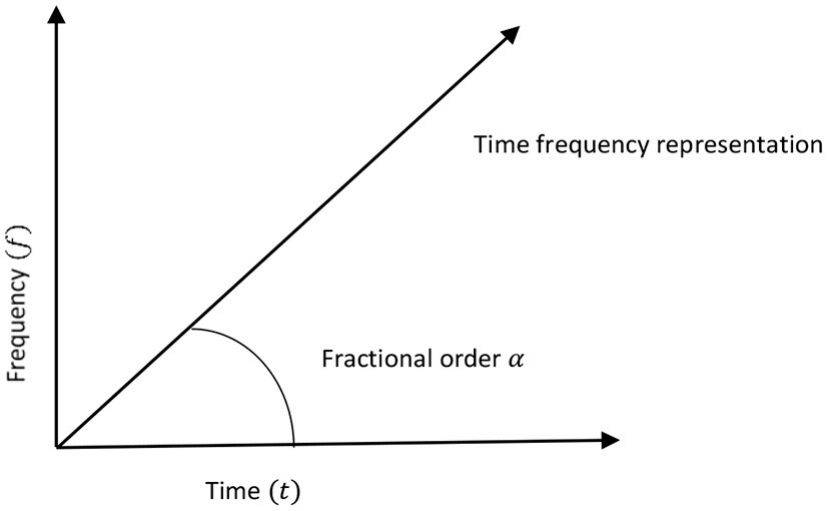
The time-frequency plane (Namias, 1980).

According to Eq. (1), the Fourier transform is a special case of the FrFT coinciding with the first order (i.e., α = 1) FrFT, whereas the zeroth order (α = 0) FrFT is the signal's representation of the space domain. The FrFT provides a joint space-frequency signal representation for all other orders. Figure 1 is a visual representation of the FrFT's functioning.

The FrFT is simple to apply successively and mathematically; the successive application of the FrFT is denoted (Ozaktas et al., 2001) as


(4) $$F_{\alpha 1}(F_{\alpha 2})=F_{\alpha 1+\alpha 2}$$

From Eq. (4), the computation of the inverse FrFT for the domain a is simply another FrFT with order −α i.e.,


(5) $$F_{-\alpha }\left(F_{\alpha }\right)=F_{\alpha -\alpha }=F_{0}=I$$

Eq. (4) and Eq. (5) are the basis for developing the watermarking system. Interestingly, a discrete representation of the FrFT exists and can be expressed in terms of the discrete Hermite-Gaussian function (Kutay et al., 1997). The mathematical representation of the discrete form of the FrFT forward kernel is given as


(6) $$F^{\alpha }\left[m,n\right]=\sum_{k=0,k\neq (N-1+{\left(N\right)}_{2})}^{N}u_{k}\left[m\right]e^{-j\frac{\pi k\alpha }{2}}u_{k}[n]$$

Where *u*_*k*_[*n*] is the *kth* discrete Hermite Gaussian function and (*N*)_2_ ≡ *N mod*2

### Blind source separation

Blind source separation refers to extracting source signals from a linear or non-linear mixture without any apriori knowledge about the sources (Yeredor, 2000). Mathematically we conceptualize the discrete linear BSS problem as


(7) X=AS

*S* is a matrix representing the collection of *N* source signals known to exist at *M* discrete points. Thus, *S* can be visualized as


(8) $$S=\left\{\begin{array}{cccc} S_{11} & S_{12} & \ldots & S_{1m}\\ \vdots & \ddots & \ddots & \vdots \\ \vdots & \ddots & \ddots & \vdots \\ S_{n1} & S_{n2} & \ldots & S_{nm} \end{array}\right\}$$

The term A in Eq. (7) is an unknown matrix of dimension *n* × *n* made of real coefficients. The mixed signals output by the mixing signals are represented by the individual rows of the matrix X. It is obvious that X is a linear mixture of all the source symbols *s*_*i*_, 1 ≤ *i* ≤ *n* and is a collection of signal mixtures observed by the receptors. The extension to the case of two-dimensional signals is straightforward. To solve the BSS problem, we must find the coefficients of the matrix S. However, in doing so, we work without any knowledge about A. Consequently, the only observed quantity available to us is matrix X. It can be observed that a solution to the linear BSS-problem reduces to finding the coefficient of the matrix A. Mathematically, it can be written as


(9) X=AS

(10) $$A^{-1}X=A^{-1}AS$$

(11) $$S=A^{-1}X$$

Thus, knowledge about the coefficients of A is sufficient to completely recover S, which is the goal of any BSS algorithm. The linear BSS problem is the one that has found the most relevance in real-world applications, though a lot of research has also focused on solving the non-linear BSS problem (Silva et al., 2020).

Various techniques have been proposed in the literature to solve the BSS problem (Song et al., 2019). Some more common approaches have focused on higher-order statistics or cumulants, the mutual information between the extracted sources, non-gaussianity of signals, principal component analysis, etc. In the present work, BSS has been used to extract the watermark from the watermarked image.

## Proposed algorithm

**Figure 2:**
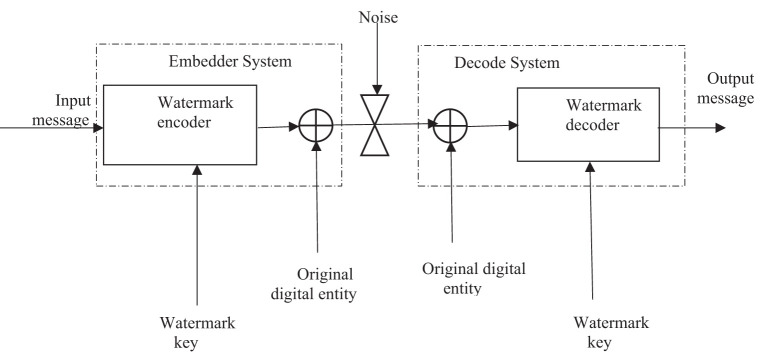
Watermark system architecture for informed watermarking (Cox et al., 2008).

Figure 2 shows the architecture of an informed watermarking system. Such systems are characterized by the fact that extraction of watermarks (Bo et al., 2011) necessitates the participation of the original watermarked digital entity or some variation. Thus, the system allows for “*source verification,”* which is an essential asset in ownership verification.

**Figure 3:**
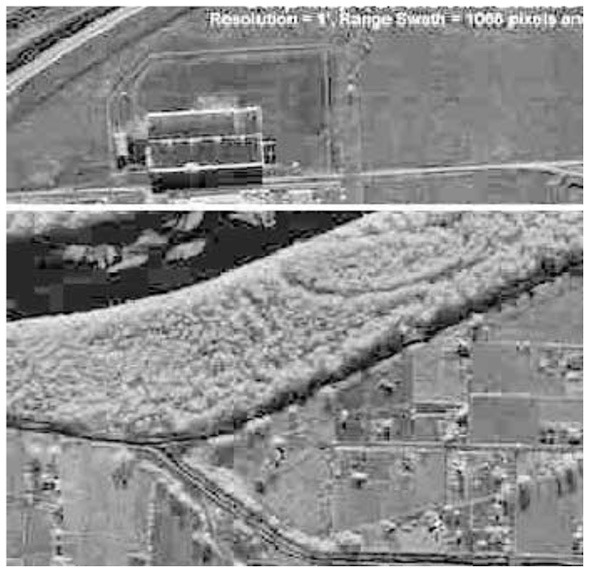
A SAR image of an agricultural field.

Figure 3 shows an example of a typical satellite image used in defense and intelligence. The image is typical as it shows several different features, all of which are distinguishable by their gray values, even though the image itself is only greyscale. The reader may also observe that the grab shows significant blocking effects and periodic noise symptoms. The common issues with images captured under non-optimal circumstances include sub-optimal environmental conditions, inappropriate lighting, acquisition device malfunction, etc. Digitally watermarking such images is a challenge due to several reasons. The induced watermark should result in a minimum variation of greyscale values of pixels, as these values are often interpreted automatically or semi-automatically. The watermark should also induce minimal distortion and artifacts, given the already impure nature of the images.

**Figure 4:**
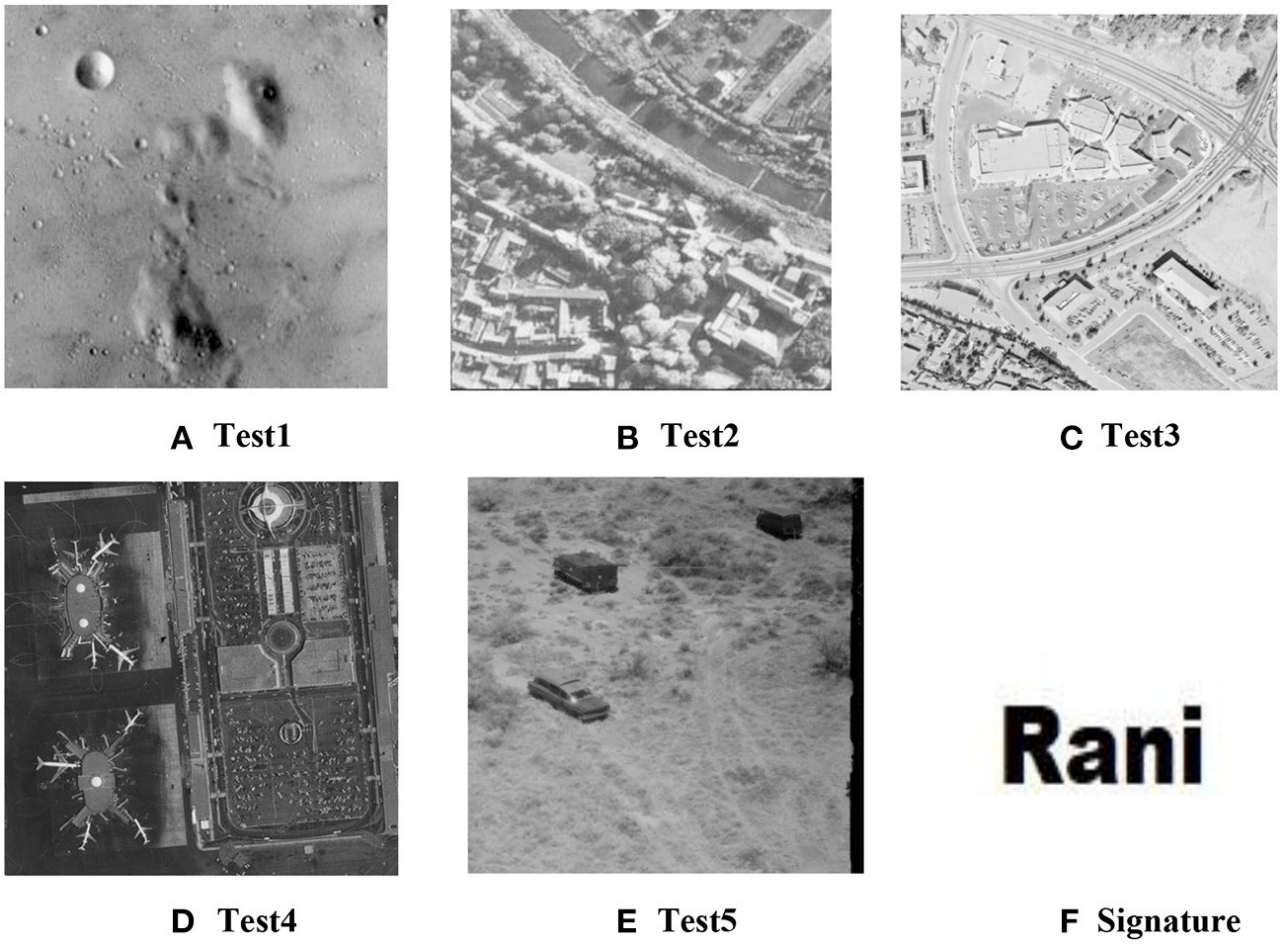
Cover images **(A–E)** and signature watermark image **(F)**.

Even though it is common to embed random patterns (usually a collection of k random numbers) as watermarks in digital images, the retrieval process is cumbersome for many applications. In high-security application domains, it makes sense to embed visually perceptible watermarks (Wang et al., 2011) that can instantly be recognized in keeping with traditional paper watermarks. Thus, the proposed method embeds binary images as watermarks in the host image. One added advantage of this is that the method can be used for steganography even though such a use cannot be recommended except for the most trivial of cases. This is because the recovered watermark may lack integrity from the original watermark, which is not desirable for a robust steganography method. Figure 4 shows some of the other test images used in this paper and the watermark that was embedded in each of these test images.

### Embedding procedure

**Figure 5:**
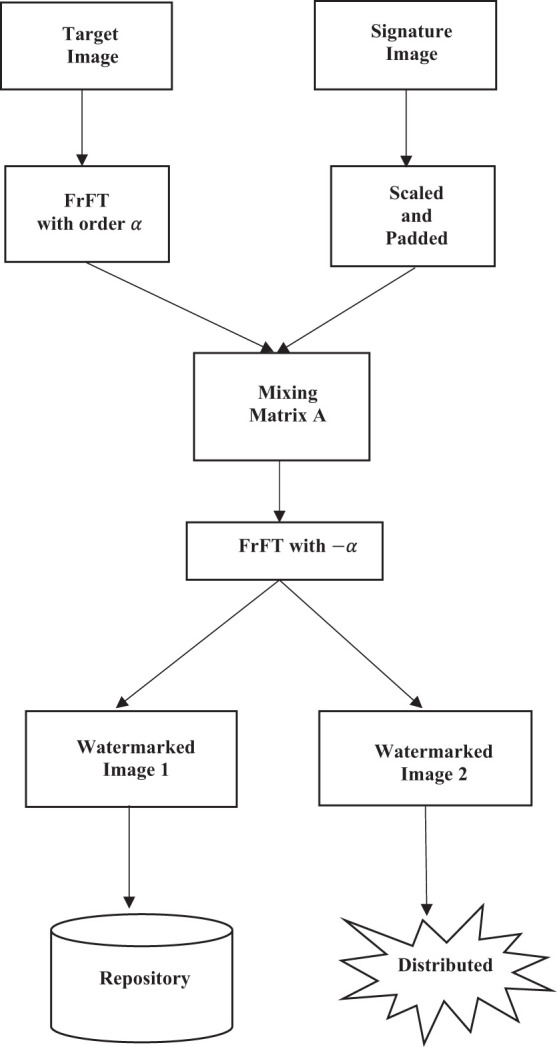
Flowchart of proposed system.

The proposed method embeds the watermark image in the α^*th*^ FrFT domain of the host image. The embedding is done with the help of a mixing matrix A of size 2 × 2, as explained in Section Blind source separation. The process of embedding the watermark is illustrated using the flowchart shown in Figure 5. The signature watermark image is padded with zeros to be the same dimension as the target or host image. In our experiments, we found that scaling the signature image's greyscale to have the same mean as the target image increases the effectiveness of the algorithm.

Embedding can be done with the watermark when the target image is first transformed into the α^*th*^ FrFT domain, and then the scaled and padded watermark image is multiplicatively introduced into the FrFT domain using a mixing matrix A. The resultant modified FrFT is returned to the spatial domain using the inverse FrFT transform, equivalent to performing an FrFT with −α. We see the resultant flowchart of watermark embedding in the target image, which found the output of two watermarked images. In an informed watermarking system, one of the resultant images is stored in a secure repository while the other can be distributed for use. By choosing a suitably high FrFT domain, the embedded watermark can be dispersed intricately in the space-frequency domain, making it very difficult to remove. In the experimental results presented, the FrFT domain chosen was α = 0.75. Mathematically the embedding process is expressed (Belouchrani et al., 1997) as


(12) $$\left[\begin{array}{c} W1\\ W2 \end{array}\right]=\;F^{-\alpha }\left\{\left[\begin{array}{c} a_{11}\&a_{12}\\ a_{21}\&a_{22} \end{array}\right]\left[\begin{array}{c} F^{\alpha }\left\{\;|f\left(x,y\right)|\;\right\}\\ \left|h\left(x,y\right)\right| \end{array}\right]\right\}$$

Where *W*1 and *W*2 are two distinct representations of the watermarked image, *a*_*ij*_ are the coefficients of the mixing matrix and |*f*(*x, y*)| and |*h*(*x, y*)| are column matrix representations of the target image and watermark image zero-padded to be of equal length. *F*^α^ is the α^*th*^ order fractional Fourier transform as discussed in section Fractional Fourier transform.

Though much research has been devoted to the possibility of using the FrFT as a tool for the digital watermarking of images (Zhang et al., 2020), not much work has focused on using the BSS in conjunction with the FrFT. An ant colony approach to optimizing the FrFT coefficients has been discussed in Al-Qaheri et al. (2010). However, using both these tools, a high level of robustness can be provided to the watermarking process. While the FrFT can diffuse the watermark over the spatial-frequency domain simultaneously, the BSS can be used to recover the watermark back with minimal effort. However, optimizing the mixing matrix to allow the embedding of an entire image requires exact tuning of the mixing matrix. One of the issues in choosing a mixing matrix-based approach to embed the watermark image in an FrFT domain is the distortion that may be induced in the watermarked image once it is brought back to the spatial domain using the inverse FrFT transform. Without any constraints to guide the choice of the coefficients of matrix A, the watermarked image usually displays many distortions. The distortions can clearly be seen in the form of wave-like structures in the top half of the image.

To reduce such distortions, the matrix coefficients must be chosen carefully. Without any apriori knowledge regarding these coefficients, the method employs the genetic algorithm to choose the optimal set of coefficients.

### Genetic algorithm-based coefficient optimization for mixing matrix

Genetic Algorithms (GAs) have long been considered an extremely efficient optimization tool for large search spaces. The functioning of these algorithms is based on the natural law of evolution and the concept of the “*survival of the fittest.”* Genetic Algorithms iterate through generations by creating a population of candidates which tend to propagate the best traits of the previous generation of candidates (Goldberg, 1989). The algorithm employs several steps to ensure that the current population converges to an optimal solution, even over large search spaces having numerous variables. The mutation operator ensures that GAs is not trapped in local maxima and can quickly converge to global solutions even in large search spaces. GAs is considered ideal for cases where a near-optimal solution has to be established in a short period.

#### Basics of genetic algorithm

1. Initialize population
2. Create initial population
3. Evaluate individuals in the initial population.
4. Create new population
5. Select-fit individuals for reproduction
6. Generate offspring with genetic operator crossover.
7. Mutate offspring.
8. Evaluate offspring.

**Figure 6:**
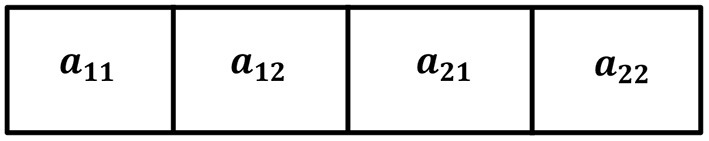
The chromosome representing the coefficients of the mixing matrix A.

In the proposed method, the four coefficients of the mixing matrix $A=\left[\begin{array}{c} a_{11}\&a_{12}\\ a_{21}\&a_{22} \end{array}\right],\;0<a_{pq}<\infty$are used to create a multi-gene chromosome for use in the GA. A typical chromosome is shown in Figure 6 (Shieh et al., 2004). The upper limit of the coefficients was restricted to 500 for experimental purposes. The fitness function used to converge the search is defined as the average RMSE of the two images in comparison to the original target image and is mathematically defined as


(13) $$F=\frac{1}{2}\left[\sum_{i=1}^{2}\sqrt{\frac{1}{MN}\sum_{x=0}^{N-1}\sum_{y=0}^{M-1}{\left[T\left(x,y\right)-\hat{T_{i}}(x,y)\right]}^{2}}\right]$$

In Eq. (13), *T*(*x, y*) represents the original image and $\hat{T_{i}}\left(x,y\right)$ represents one of the watermarked images. The images are considered of size *M* × *N* for generality, but in our experiments, only square images were used for the sake of computational simplicity. Using the GA-based approach (Wang et al., 2011), the mixing matrix used to embed the watermark produced minimal distortions in the watermarked images when using reasonably small-sized watermarked images. However, the distortions show a marked increase when the size of the watermark image is increased relative to the target image. As the sole aim of the signature image is to verify the authenticity and ownership of the target image, even small watermarks are more than sufficient for this purpose.

**Figure 7:**
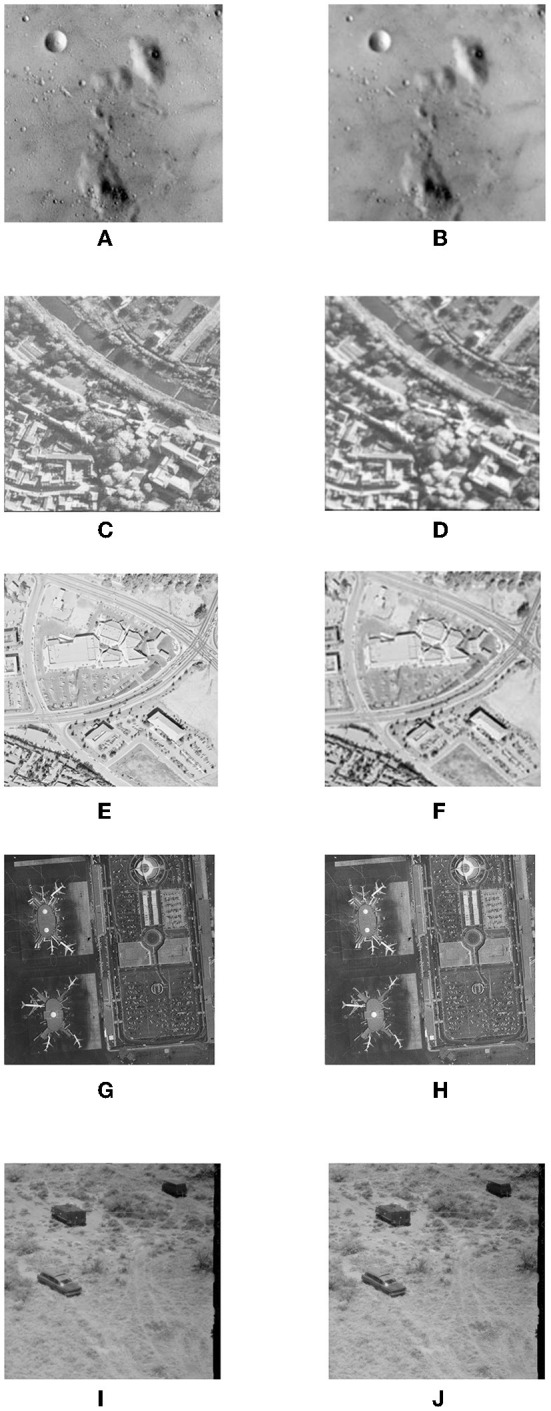
Watermarked images using GA-based mixing matrix coefficient optimization and its objective evaluation of imperceptibility for different images. **(A)** Original Test1. **(B)** Watermarked Image (PSNR = 51.2 and SSIM = 0.98). **(C)** Original Test2. **(D)** Watermarked Image (PSNR = 53.9 and SSIM = 0.95). **(E)** Original Test3. **(F)** Watermarked Image (PSNR = 53.9 and SSIM = 0.96). **(G)** Original Test4. **(H)** Watermarked Image (PSNR = 50.6 and SSIM = 0.96). **(I)** Original Test5. **(J)** Watermarked Image (PSNR = 53.8 and SSIM = 0.96).

Figures 7B,D,F,H,J shows all watermarked images using the GA-based optimization method. The lack of wave-like distortions in the top half of the image is noticeable (compared with Figures 7A,C,E,G,I respectively). It is evident that the watermarked image is heavily distorted for any signature image larger than approximately 30% of the size of the target image. Very slight wave-like disturbances in the image are noticeable toward the top left corner of the image. However, they are quite insignificant in the context of the overall image.

### Watermark extraction

**Figure 8:**
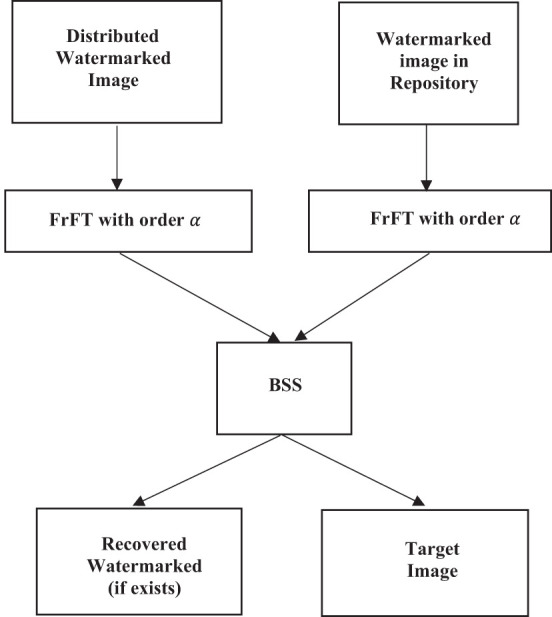
Schematic representation of watermark extraction.

The watermark extraction in the proposed method is straightforward to perform. The process inverts the extraction method and is depicted using the flowchart shown in Figure 8. It is interesting to note that the actual extraction employs the technique of BSS, and the knowledge of the mixing matrix A used at the encoding end is not required during the retrieval process. The FrFT domain α in which the watermark was embedded functions as the key in the watermarking scheme, as shown in Figure 2. Our experimental setup used the highly efficient FASTICA package to perform the BSS. **Figure 10** shows the recovered watermark using the retrieval process. Even though traces of the frequency component of the target image are visible in the retrieved watermark, the extracted watermark has been extracted with remarkable clarity.

**Figure 9:**
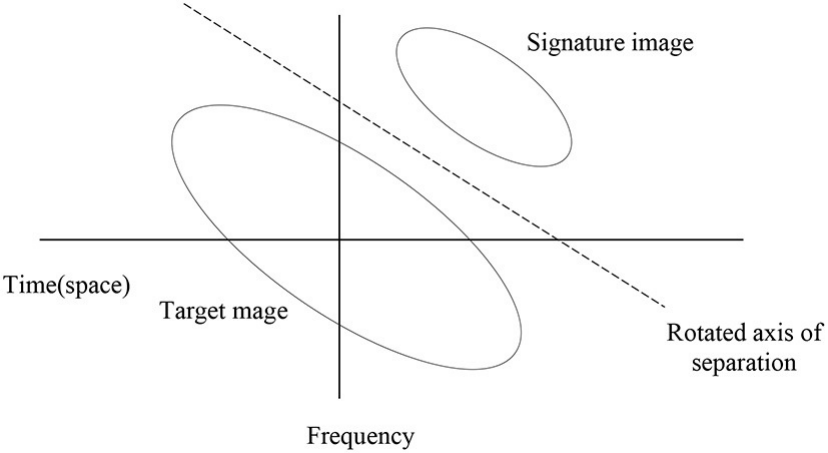
A view of the Wigner's plane in the watermarking scheme.

Due to its time (space) frequency capabilities embedding watermarks in the FrFT domains is a highly robust way of securing images. Figure 9 shows a visual representation of watermark embedding in the FrFT domain. The figure shows the target and signature images in the Wigner plane (Xia et al., 1997), with the two axes representing space and frequency, respectively. It is seen that the watermark image cannot be separated from the target image in either the spatial domain or the frequency domain alone. Thus, targeted attacks to remove the watermark in any of these domains are not likely to succeed, and this significantly increases the robustness of the method. Figure 9 also shows that the oblique FrFT axis, which corresponds to a rotation in the Wigner plane, can separate the two components, and it is in this domain that we perform BSS to extract the watermark. Thus, knowledge of the correct FrFT domain is essential in extracting the watermark. **Figure 13** shows the PSNR of the recovered watermark for different FrFT domains. The actual embedding of the watermark was performed in the FrFT domain α = 0.75. The plot clearly shows that the watermark has failed to be extracted for all other domains except for the domain in which it was embedded.

## Experimental results and discussions

To evaluate the robustness of the proposed watermarking method, the watermarked images were subjected to several common signal processing attacks (Lu et al., 2003). Due to the visual nature of the embedded watermark, the quality of the retrieved watermark is subject to human interpretation rather than statistical parameters, e.g., PSNR (Kumari and Mustafi, 2021) and RMSE (Alvarez et al., 2018). This is often an advantage for end-users. The experimental results show that the watermark can be successfully extracted at the retrieval end in almost all cases.

**Figure 10:**
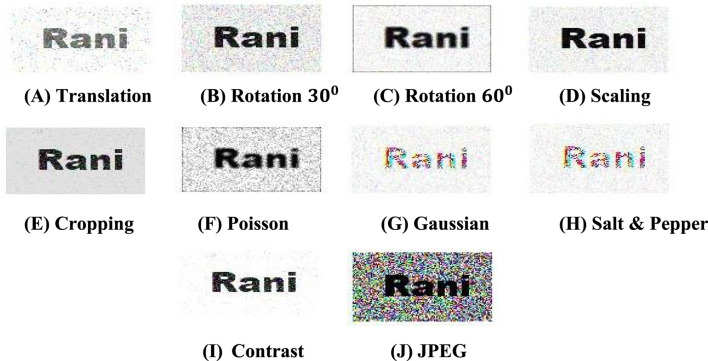
Schematic representation of extracted watermark after performing some geometric attacks. **(A)** Translation. **(B)** Rotation 30^0^. **(C)** Rotation 60^0^. **(D)** Scaling. **(E)** Cropping. **(F)** Poisson. **(G)** Gaussian. **(H)** Salt and Pepper. **(I)** Contrast. **(J)** JPEG.

Figure 10 summarizes the performance of the method for various test cases of simulated attacks. In Figure 10 results, the watermarked image has been blurred using a Gaussian filter (Zhang et al., 2020). Such filters are very mild and do not have a significant abrasive effect on the image. The recovered watermark shows a high degree of clarity, as seen in Figure 10G. However, the watermark is still reasonably extracted and is visually recognizable. A similar result is observed in the case of salt and pepper noise. The watermark is still perceptible even though salt and pepper noise distorts the entire frequency spectrum and often adversely affects many watermarking schemes. In Figure 10E, the watermarked image shows a simulated cropping effect, replacing a section of the image with black grayscale values. The crop position is intentionally chosen as standard embedding using a mixing matrix that would place the watermark at the top left of the target image. Even though the recovered watermark shows marked distortions, it is quite recognizable even by the naked eye, and the authenticity of the image can be validated.

As can be seen, the method is quite robust and can detect the watermark in the case of most attacks. In a few cases, like Gaussian filtering and JPEG compression (Naheed et al., 2014), the choice of the FrFT domain affected the performance of the method, and the retrieval was found to be more efficient in FrFT domains.

The proposed method can easily be extended to multiple plane formats like RGB images, where two alternative methods for embedding the watermark may be adopted. The watermark may be embedded in one of the three planes (which increases the robustness of the algorithm to a small extent), or the watermark image can be partitioned and embedded in all three planes. The second method is interesting as the choice of the FrFT domain α can be different for the three planes. Another possible improvement can be to choose an optimal FrFT domain to embed the watermark. Our experiments observed that the best results were obtained for the higher FrFT domains, but some domains performed better than others. The choice of the FrFT domain can again be performed using a heuristic or meta-heuristic algorithm, e.g., GA. However, even for randomly chosen non-optimized FrFT domains, the method is found to be highly competent.

## Performance evaluation criteria

Imperceptibility, robustness, payload, and security are four attributes that determine the quality of an image watermarking scheme (Fares et al., 2020). Further, the algorithmic complexity is also often considered an important parameter while judging the efficiency of a watermarking algorithm.

### Quality metrics

While the quality of an image watermarking scheme can be judged by the human visual system (HVS) using our latent sense of perception (Shih, 2017), several mathematical techniques have been suggested in the literature to measure the performance of an image watermarking scheme quantitatively.

In the current work, we have employed four conventional performance metrics to evaluate the imperceptibility and robustness of the proposed algorithm. These quality metrics (Woods and Gonzalez, 2002) are peak signal-to-noise ratio (PSNR), structural similarity index measurement (SSIM), normalized cross-correlation (NC), and bit error rate (BER). Among these, PSNR and SSIM have been used to evaluate the imperceptibility of a digital watermark, while NC and BER test the robustness of the proposed method. A brief description of these quality metrics is provided in the following sections.

### Imperceptibility and capacity test

**Figure 11:**
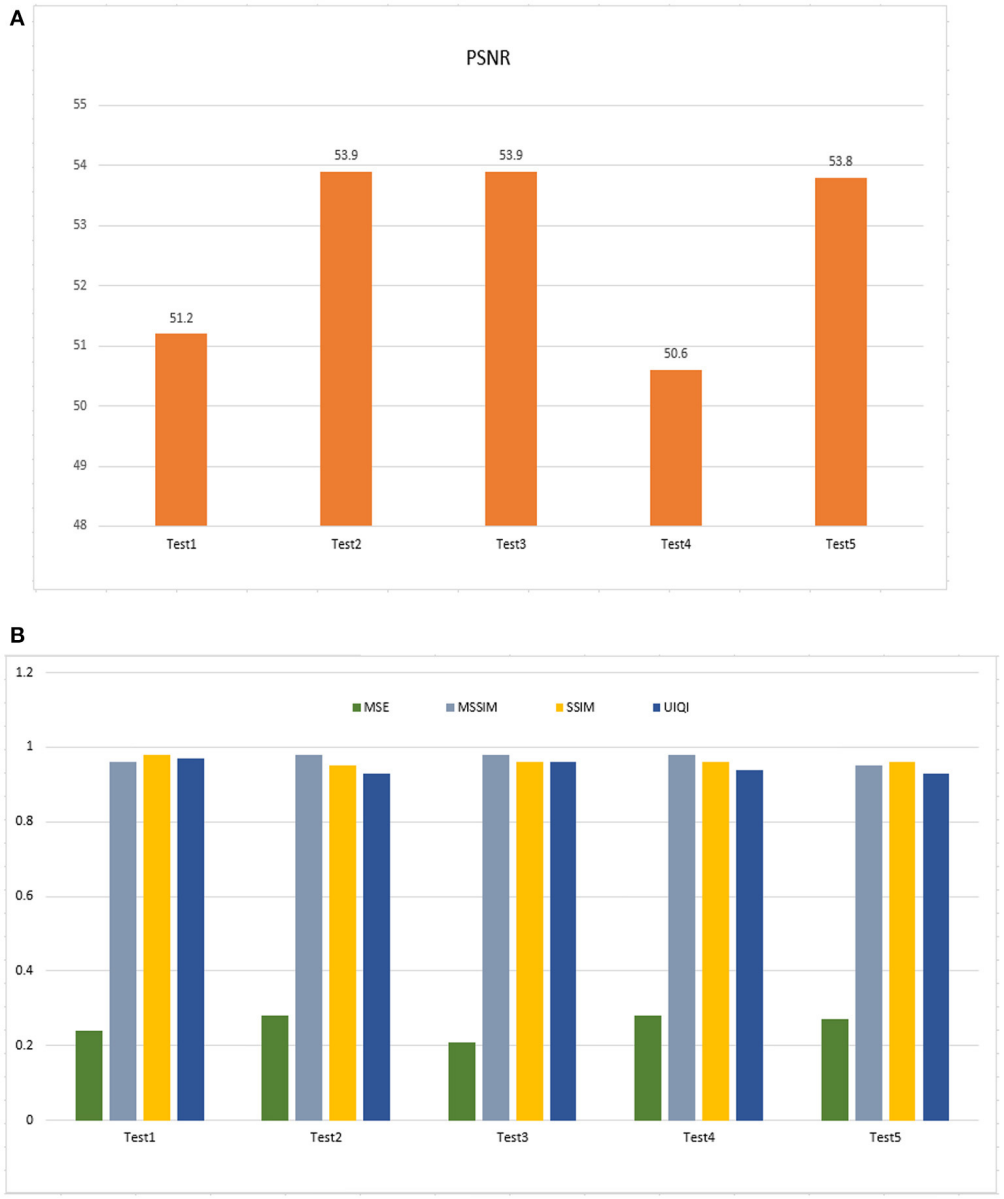
**(A)** Performance outcome of imperceptibility (PSNR). **(B)** The outcome of MSE (Mean Square Error), MSSIM (Mean Structure Similarity Index Measure), SSIM (Structure Similarity Index Measure), and UIQI (Universal Index, Quality Index).

The tests outlined in the previous section were performed to evaluate the proposed method. Figure 11A shows PSNR values for experimental images. PSNR readings remain high, proving the watermark is imperceptible. Figure 11B shows MSE values between 0.21 and 0.28, indicating a minimal loss in watermarked image quality. Maximum UIQI values are close to 1 (0.93–0.97). This illustrates that watermarked images always seem to be like the originals. Regarding structural similarity, the original and watermarked are comparable, and the highest value for both SSIM and MSSIM is 0.98, indicating high perceptual quality.

### Robustness test

**Figure 12:**
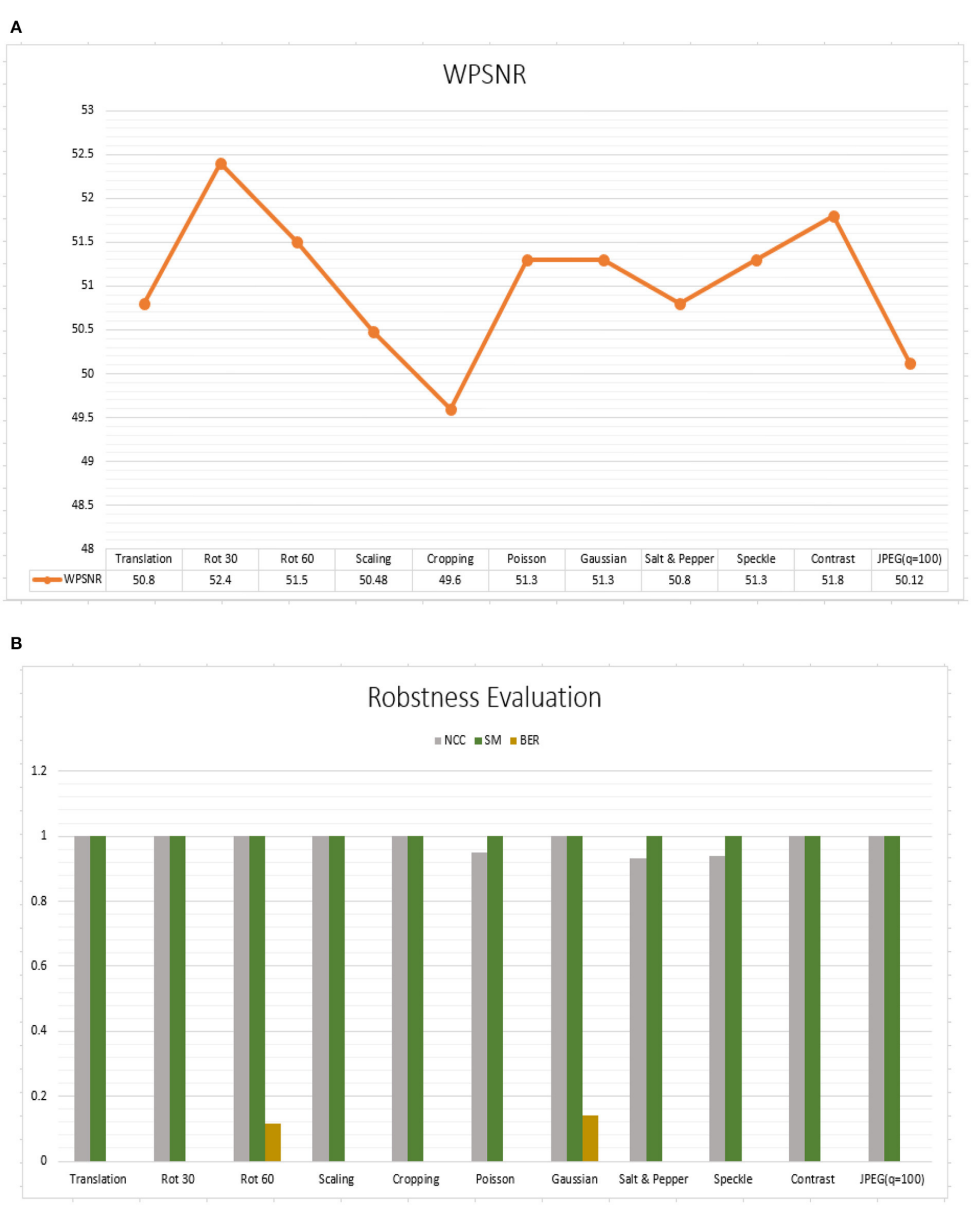
**(A)** Performance outcome of robustness (WPSNR), **(B)** Performance outcome of NCC (Normalized Cross-Correlation), SM (Similarity Measurement), BER (Bit Error Rate).

Robustness can be determined by examining the extracted watermark after the watermarked image has been attacked (Shih, 2017). We evaluated the algorithm against geometric attacks. Table 1 depicted the watermark and extracted the watermark's robustness after some attacks. In Figure 12A, we had shown the WPSNR values for the experiment conducted. In Figure 12B, performance outcome of NCC (Normalized Cross-Correlation), SM (Similarity Measurement), BER (Bit Error Rate) has been shown. The table demonstrates that the maximum WPSNR value is 52db which is good. NCC results are also excellent, except for Poisson, Salt & Pepper, and Speckle. NC > 0.93 means the original and extracted watermarks are similar. The SM (Similarity Measurement) also reports promising findings. Except for Rotation and Gaussian noise addition, the Bit Error Rate is less. The proposed algorithm is resilient against attacks.

**Table 1:** Evaluation of different geometric attacks with their respective robustness results.

**Attacks type**	**WPSNR**	**NCC**	**SM**	**BER**
Translation	50.8	1	1	0
Rotation **30**^**0**^	52.4	1	1	0
Rotation **60**^**0**^	51.5	1	1	0.114
Scaling	50.48	1	1	0
Cropping	49.6	1	1	0
Poisson	51.3	0.95	1	0
Gaussian	51.3	1	1	0.14
Salt and pepper	50.8	0.93	1	0
Speckle	51.3	0.94	1	0
Contrast	51.8	1	1	0
JPEG (q = 100)	50.12	1	1	0

**Figure 13:**
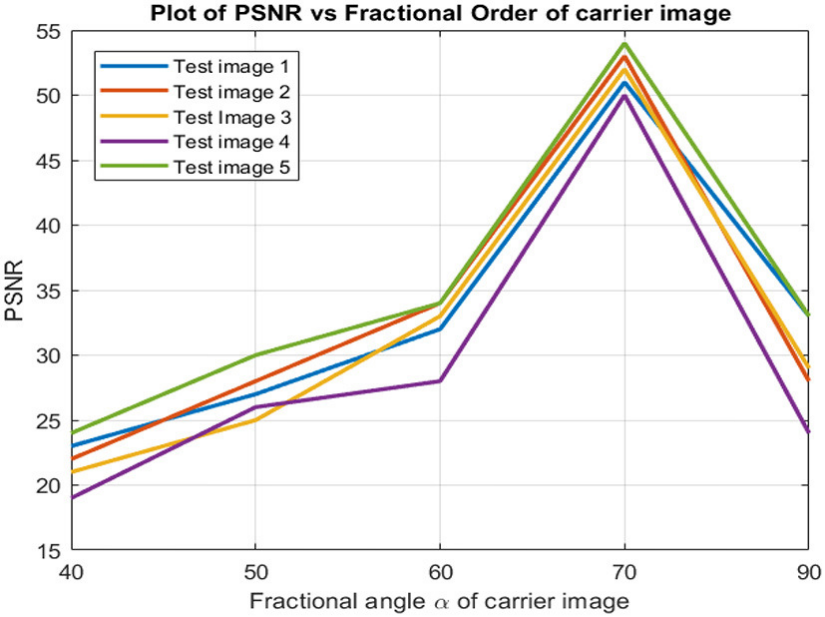
PSNR results of images with different fractional order α.

The PSNR values acquired across various fractional orders have also been evaluated, and we found that, most often, the best PSNR was obtained almost at a rotation angle of 30°. In Figure 13, the PSNR value of the watermarked image gradually decreased on each side. The optimal embedding, according to this, occurs in the higher fractional orders. The optimal embedding fractional order had to be determined manually for each image, resulting in one of the drawbacks of the present work. This is an additional computing load, and research efforts may be directed toward developing more effective techniques for determining the appropriate fractional order.

## Conclusion

In this paper, a novel informed watermarking technique for digital images has been proposed. The method uses the fractional Fourier transform and BSS to embed and extract the watermark. The embedded watermark is intentionally chosen to be visually recognizable to make the retrieval and identification process more conducive for typical end-users. The method also utilizes GA to optimize the embedding phase, ensuring that the watermark can be embedded in the target image with minimum distortions. Further work to provide RST invariance to the method only make the technique more robust. Currently, the usefulness of the Log polar transform is being explored to provide the necessary RST invariance property to the method. Additionally, more research must be conducted to optimize the process more robustly when faced with significant image cropping, in which case the watermark recovery is significantly hampered.

According to the results presented, the method works highly efficiently for the average case and is also very robust against many known signal processing attacks. Another important consideration while evaluating the algorithm's robustness is that the retrieval process depends stringently on correctly identifying the FrFT domain. For all other domains, the watermark stays hidden and thus does not lend itself to passive attacks or masquerades.

## Data availability statement

The original contributions presented in the study are included in the article, further inquiries can be directed to the corresponding authors.

## Author contributions

RK: original draft writing, conceptualization, methodology, validation, and software. AM: conceptualization, methodology, validation, research, writing-review and editing, and supervision. All authors contributed to the article and approved the submitted version.

## Conflict of interest

The authors declare that the research was conducted in the absence of any commercial or financial relationships that could be construed as a potential conflict of interest.

## Publisher's note

All claims expressed in this article are solely those of the authors and do not necessarily represent those of their affiliated organizations, or those of the publisher, the editors and the reviewers. Any product that may be evaluated in this article, or claim that may be made by its manufacturer, is not guaranteed or endorsed by the publisher.
